# Editorial comment: Is testosterone replacement an effective treatment of secondary premature ejaculation?

**DOI:** 10.1590/S1677-5538.IBJU.2021.03.07

**Published:** 2021-03-25

**Authors:** Valter Javaroni

**Affiliations:** 1 Hospital Federal do Andaraí Departamento de Andrologia Rio de JaneiroRJ Brasil Departamento de Andrologia, Hospital Federal do Andaraí, Rio de Janeiro, RJ, Brasil

İbrahim Nuvit Tahtali ^1^

Andrologia. 2020 Feb;52(1):e13452.

## COMMENT

Although it is well established that all the aspects of male reproduction are hormonally regulated, the endocrine control of the ejaculatory reflex is still not completely clarified (1).

It has been proposed that gonadal, thyroid and pituitary hormones (oxytocin and prolactin - PRL) control, at various levels, the ejaculatory process and its overall latency time. We already know that hyperthyroid patients had more chance to suffer premature ejaculation (PE), a prevalence that could be substantially reduced by treating the underlying disease, with a consequent doubling of ejaculatory latency (2).

The ejaculation distribution theory by Waldinger (3) postulates that IELT in men is represented by a biological continuum with men with PE belonging to the extreme left side of the intravaginal ejaculation latency time (IELT) curve. It is postulated that any random sample of men is likely to include a minority of men with (nearly) always early ejaculation, a second minority who (nearly) always suffer from a delayed or failure of ejaculation, while the majority have a ‘‘normal’’ or ‘‘average’’ ejaculation time. This biological variation may be due, at least in some cases, to a different regulation of the serotonergic receptor subtypes (3).

The levels of the three hormones: PRL, testosterone (T) and thyrotropin (TSH) were correlated in a survey among more than 2000 men, where 674 (25.2%) and 194 (7.3%) reported premature and delayed ejaculation, respectively. While PRL as well as TSH levels progressively increased from patients with severe PE towards those with anejaculation, the opposite was observed for testosterone levels. The observational nature of this study does not allow to directly support a causal role for any of these hormones in determining the ejaculatory latency, but the results support the concept of an endocrine regulation of ejaculation besides other aspects of the male sexual response (1).

Therefore, T is one endocrine factor that has been involved in the control of ejaculation. Expression of androgen receptors in smooth muscles of the male genital tract, in the spinal nucleus of the bulbocavernosus muscle, and in the medial preoptic area of the hypothalamus all suggest that androgens facilitate ejaculatory response at different levels (4).

The reasons of the association between T and ejaculation are not easy to explain. However, some hypotheses could be drawn. The first explanation is psycho-endocrine: T level, besides its action on sexual response, profoundly influences male behavior. High T level in human adult is associated with leadership, toughness, personalized power, and aggressive dominance. It could be speculated that hypogonadism and its related reduction in sexual confidence and related aggressiveness could play a critical role in the control of ejaculation timing, reducing the internal cues for reaching orgasm and ejaculation (5).

The second hypothesis is neurological: some data from animal model seem to support a central action of T in the control of the ejaculation reflex. In particular, Keleta et al. (6) demonstrated that the chronic treatment with T in rats significantly decreased 5-hydroxytryptamine in brain pointing an interesting association with Waldinger’s neurobiological theory.

Another intriguing explanatory option is about a possible peripheral role of T in regulating male genital tract motility. The integrated system nitric oxide- phosphodiesterase type 5 (PDE5), one of the most important factors involved in the contractility, is under T control. In an experimental model of hypogonadotropic hypogonadism, was found that in vas deferens of T-deficient rabbits cGMP degradation was reduced and PDE5 less expressed and biologically active. T administration completely reverted these alterations (7).

However the role of T in pathogenesis of ejaculatory symptoms has not been completely clarified. Some reports suggest that low serum T levels may contribute to ejaculatory dysfunction - the ejaculatory dysfunction consists in at least three separate symptoms: PE; delayed ejaculation (DE) and anejaculation; and the retrograde ejaculation - and those men with androgen deficiency are twice as likely to experience this condition (8). Low serum T levels have been inconsistently associated with PE, while others’ reports suggested that hypogonadism can be considered a possible cause of DE (9, 10). The majority of studies have shown an increase in the levels of testosterone in men with PE (11).

Ejaculatory dysfunction can also be coupled to other sexual disturbances. The results of large surveys demonstrated that PE is suffered by up to 50% of patients with erectile dysfunction, which again points to a complex mechanism between testosterone levels, ejaculation and erectile function (12).

With the aim of evaluate the possible contribution of T and hypogonadism in the control of the ejaculatory reflex, Corona G. et al. (13) in 2008 compared subjects with PE or DE to those without ejaculatory dysfunction. In the youngest age band (25–40 years), subjects with PE reported higher T levels when compared to the other groups (subjects with DE or those without PE and DE). Conversely, in the oldest age band (55–70 years), lower T levels were observed in DE subjects. Accordingly, patients with PE showed the lowest and subjects with DE the highest prevalence of hypogonadism. These differences were confirmed even after adjustment for confounders such as age and libido (13).

Given the lack of a consensus definition of DE and the paucity of epidemiologic and prevalence data, men with ejaculatory complaints other than PE can pose a challenge to the sexual health practitioner. Based on some evidence of lower T levels in men with DE, the hypothesis - T replacement could offer benefit in androgen-deficient men (total T levels <300 ng/dL) with ejaculatory dysfunction defined as delayed ejaculation, anejaculation, decreased force of ejaculation, or decreased ejaculate volume – was tested in a randomized trial where the primary outcome was a change in the score of the three-item Male Sexual Health Questionnaire-Ejaculatory Dysfunction-Short Form (MSHQ-EjD-SF). T replacement was not associated with significant improvement in ejaculatory dysfunction in androgen deficient men (14).

In another study with a large sample of community-dwelling men with ejaculatory complaints, an analysis was done to examine the self-reported intravaginal ejaculatory latency time (IELT) and masturbatory ejaculation latency times in men claiming to have DE vs men without DE. A second objective was to examine hormonal and demographic predictors of ejaculation time in the DE vs non-DE cohorts. This study proved that men with DE complain really had an increased IELT but failed to find that men reporting DE had lower T concentrations than men not reporting DE (15).

On another hand, one observational survey found no correlation in men with PE diagnosed by Premature Ejaculation Diagnostic Tool (16) (PEDT) with T level, but author’s identified luteinizing hormone level (OR, 1.293; p=0.014) as an independent risk factor for premature ejaculation (17).

And finally we arrived at this 2020 publication. Although as we could see the majority of studies on this topic have suggested that patients with PE have higher T levels than those without (11), this publication focuses on T replacement therapy in secondary premature ejaculation patients with T deficiency.

All patients’ International Index of Erectile Function-5 (IIEF-5) scores, IELT via stopwatch and PEDT scores were assessed and recorded at baseline and after 6 months of interventional therapies.

Patients with secondary PE, which diagnoses were based on the definitions of PE put forth by the International Society for Sexual Medicine (ISSM) and were confirmed by utilization of the PEDT, who consistently had testosterone levels under 280 ng/dl in all three initial measurements within the first month of study inclusion, received testosterone replacement therapy every 2 weeks (n = 20), while 28 patients with secondary PE received dapoxetine.

The median (min–max) IELT of those who received testosterone had increased to 120 (60–300) seconds from a pre-treatment value of 15 (0–90) seconds (p < 0.001). In dapoxetine recipients, median IELT was increased to 70 (0–240) seconds from a pre-treatment value of 60 (0–120) seconds (p < 0.001). Both treatment groups also showed improvements in post-treatment PEDT scores that was statistically significant in only those who received testosterone (p = 0.002 in the testosterone group, p = .062 in the dapoxetine group).

Recognizing that the study has some important limitations such as no randomization; open label; lack of a control group; the absence of an arm with T plus dapoxetine; the use of PEDT and IELT as measurements of efficacy instead of restoration of ejaculatory control and increasing in sexual satisfaction as better markers of treatment success; and mainly the small sample size; authors concluded that their patients with hypogonadism and secondary PE had greater benefit from T replacement than with dapoxetine.

Together with other evidences, including the already mentioned failure in a randomized trial to improve ejaculation with T replacement in hypoganadal men with DE (14), this recent publication suggests the existence of a more complex and indirect relationship between T level and ejaculatory control. This is an area of knowledge that needs more robust studies and represents a great opportunity for good researchers.
